# Ghrelin at the interface of hunger, reward and obesity

**DOI:** 10.1192/j.eurpsy.2023.34

**Published:** 2023-07-19

**Authors:** S. L. Dickson

**Affiliations:** Institute of Neuroscience and Physiology, The Sahlgrenska Academy at the University of Gothenburg, Gothenburg, Sweden

## Abstract

**Abstract:**

Ghrelin, a stomach-derived hunger and appetite signal, drives behaviors that ensure we seek out and consume foods, not only in situations of energy deficit but also when anticipating palatable foods. Key target pathways for ghrelin include the orexigenic agouti-related peptide (AgRP) neurones in the hypothalamic arcuate nucleus (Arc), that appear to confer the unpleasant feelings of hunger. They also include dopamine neurones in the ventral tegmental area, where ghrelin heightens motivation for food rewards. Recently, we have employed a variety of neural circuit mapping techniques in rodents to help clarify the function of populations of ghrelin-responsive targets. We found that 1) chemogenetic activation of ghrelin-responsive cells in the Arc is sufficient to drive a feeding response and to induce food-motivated behaviour and 2) that dopamine neurones in the VTA are activated when mice are exposed to cues that predict a food reward than to its retrieval, as revealed by fiber photometry recordings from these cells. Further studies aim to determine the role of ghrelin-responsive cells in the parabrachial nucleus of the brainstem. Overall, the brain ghrelin signalling system is well positioned to integrate the response to hunger, enhanced by both intrinsic and external cues and in ways that are relevant to curb over-eating in obesity.

**Disclosure of Interest:**

None Declared

